# Supplementary material for: OpenMM 7: Rapid development of high performance algorithms for molecular dynamics
Source: PLoS Comput Biol. 2017 Jul 26;13(7):e1005659. doi: 10.1371/journal.pcbi.1005659 (PMC5549999; doi:10.1371/journal.pcbi.1005659)
Supplement: S1 Source Code — (ZIP) [file pcbi.1005659.s002.zip › OpenMM-7.0.1-Source/docs/api-python/generated/simtk.openmm.openmm.SerializationProxy.html]

SerializationProxy — OpenMM 7.0.1.dev-5e86c4f documentation


# SerializationProxy¶

*class* `simtk.openmm.openmm.``SerializationProxy`(*\*args*, *\*\*kwargs*)¶
:   A SerializationProxy is an object that knows how to serialize and deserialize objects of a particular type. This is an abstract class. Subclasses implement the logic for serializing particular types of logic.

    A global registry maintains the list of what SerializationProxy to use for each type of object. Call registerProxy() to register the proxy for a particular type. This is typically done at application startup or by a dynamic library’s initialization code.

    `__init__`(*\*args*, *\*\*kwargs*)¶

    Methods

    |  |  |
    | --- | --- |
    | `__init__`(\*args, \*\*kwargs) |  |
    | `deserialize`((self, node) -> void \*) | Reconstruct an object from its serialized data. |
    | `getProxy`((typeName) -> SerializationProxy) | getProxy(type) -> SerializationProxy |
    | `getTypeName`((self) -> std::string const &) | Get the name of the object type this proxy manipulates, as passed to the constructor. |
    | `registerProxy`(type, proxy) | Register a SerializationProxy to be used for objects of a particular type. |
    | `serialize`(self, object, node) | Subclasses implement this method to record information about an object being serialized. |

    *static* `registerProxy`(*type*, *proxy*)¶
    :   Register a SerializationProxy to be used for objects of a particular type.

        |  |  |
        | --- | --- |
        | Parameters: | - **type** (*type\_info*) – the type\_info for the object type - **proxy** (*SerializationProxy \**) – the proxy to use for objects of the specified type |

    *static* `getProxy`(*typeName*) → SerializationProxy¶
    :   getProxy(type) -> SerializationProxy

        Get the SerializationProxy to use for objects of a particular type, specified by type\_info.

        |  |  |
        | --- | --- |
        | Parameters: | **type** (*type\_info*) – the type\_info of the object type to get a proxy for |

    `getTypeName`(*self*) → std::string const &¶
    :   Get the name of the object type this proxy manipulates, as passed to the constructor.

    `serialize`(*self*, *object*, *node*)¶
    :   Subclasses implement this method to record information about an object being serialized.

        |  |  |
        | --- | --- |
        | Parameters: | - **object** (*void \**) – a pointer to the object being serialized - **node** (*SerializationNode*) – all data to be serialized should be stored into this node, either directly as properties or indirectly by adding child nodes to it |

    `deserialize`(*self*, *node*) → void \*¶
    :   Reconstruct an object from its serialized data.

        |  |  |
        | --- | --- |
        | Parameters: | **node** (*SerializationNode*) – a SerializationNode containing the object’s description |
        | Returns: | a pointer to a new object created from the data. The caller assumes ownership of the object. |
        | Return type: | void \* |

    `__delattr__`¶
    :   x.\_\_delattr\_\_(‘name’) <==> del x.name

    `__format__`()¶
    :   default object formatter

    `__getattribute__`¶
    :   x.\_\_getattribute\_\_(‘name’) <==> x.name

    `__hash__`¶

    `__reduce__`()¶
    :   helper for pickle

    `__reduce_ex__`()¶
    :   helper for pickle

    `__sizeof__`() → int¶
    :   size of object in memory, in bytes

    `__str__`¶

High performance molecular simulation on GPUs


### Navigation

- Application Layer
- Library Layer
  - Core Objects
  - Forces
  - Integrators
  - Extras

©2015, Stanford University and the Authors.
|
Powered by Sphinx 1.3.1
& Alabaster 0.7.7
|
Page source
